# Mass balance, excretion, and pharmacokinetics of a single oral dose of [^14^C]pixavir marboxil in healthy Chinese male participants

**DOI:** 10.3389/fphar.2026.1905373

**Published:** 2026-08-11

**Authors:** Chunyang Zhao, Ying Yang, Gangyi Liu, Lin Qian, Yun Liu, Cheng-Yuan Tsai, Li-Wen Chang, Qian Chen, Hongjie Qian, Jingying Jia, Yanmei Liu

**Affiliations:** 1 Drug Clinical Trial Center, Shanghai Xuhui Central Hospital/Xuhui Hospital, Fudan University, Shanghai, China; 2 Shanghai Engineering Research Center of Phase I Clinical Research and Quality Consistency Evaluation for Drugs, Shanghai, China; 3 Joincare Pharmaceutical Group Industry Co., Ltd., Shenzhen, Guangdong, China; 4 TaiGen Biotechnology Co., Ltd., Taipei, Taiwan

**Keywords:** cap-dependent endonuclease inhibitor, excretion, healthy participants, mass balance, metabolite identification, pharmacokinetics, pixavir marboxil, TG-1000

## Abstract

**Background and Objective:**

Cap-dependent endonuclease (CEN) inhibitors are a promising novel class of antiviral agents for influenza treatment. This phase I study aimed to evaluate the mass balance, excretion pathways, and pharmacokinetics of pixavir marboxil (TG-1000), a novel CEN inhibitor prodrug, in healthy humans.

**Methods:**

Six healthy Chinese male participants received a single oral dose of 40 mg (100 μCi) [^14^C]pixavir marboxil. Blood, urine, and fecal samples were collected at predefined intervals up to 384 h post-dose. Total radioactivity was determined using oxidative combustion and liquid scintillation counting. Pharmacokinetic parameters were calculated using non-compartmental analysis, and metabolite profiling was conducted using high-performance liquid chromatography coupled with radiochemical detection and high-resolution mass spectrometry.

**Results:**

Intact pixavir marboxil was undetectable in plasma. Systemic exposure primarily consisted of its active metabolite, pixavir (TG-0527), and a subsequent glucuronide conjugate, TG-0600771. The geometric mean areas under the curve ( ${AUC}_{0-\infty }$ ) for total radioactivity, pixavir, and TG-0600771 in six participants were 4,220 hng Eq./mL, 2,490 hng/mL, and 981 hng/mL, respectively. The administered radioactive dose demonstrated a mean cumulative recovery of 91.29%, with 85.62% recovered in feces and 5.68% in urine. Metabolite profiling identified pixavir and TG-0600771 as the major metabolites, with no major cytochrome P450 (CYP)-mediated oxidative metabolites observed in plasma. The single dose of [^14^C]pixavir marboxil was safe and well-tolerated.

**Conclusion:**

Pixavir marboxil exhibits a favorable pharmacokinetic profile characterized by rapid systemic conversion to its active metabolite, CYP-independent metabolism, and predominantly non-renal clearance. These favorable pharmacokinetic characteristics support a well-characterized disposition profile with limited reliance on CYP-mediated metabolism and renal excretion of active pixavir.

**Trial Registration:**

This study was registered with Drug Clinical Trial Registration and Information Disclosure Platform, National Medical Products Administration (NMPA), number CTR20231822 (http://www.chinadrugtrials.org.cn/).

## 1 Introduction

Seasonal influenza is a contagious respiratory viral infection that drives recurrent epidemics, imposing a significant global burden of morbidity and mortality (Kyaw et al., 2024; Shao et al., 2026). For decades, neuraminidase inhibitors (NAIs) such as oseltamivir have been widely used as the primary antiviral therapy. However, their real-world clinical utility is frequently limited by a narrow therapeutic window—requiring initiation within 48 h of symptom onset to maximize clinical benefit—along with the persistent risk posed by circulating drug-resistant viral strains (Muthuri et al., 2014; Zhang et al., 2022; Patel et al., 2024). Consequently, there is an urgent clinical need to develop novel antiviral strategies with broader therapeutic windows and higher barriers to resistance.

Cap-dependent endonuclease (CEN) inhibitors represent a novel therapeutic class that offers a promising strategy to overcome these clinical challenges (Yang, 2019). By selectively targeting the highly conserved polymerase acidic (PA) subunit of the viral RNA polymerase complex, CEN inhibitors effectively halt viral mRNA transcription at an earlier stage of the replication cycle compared to NAIs (Fukao et al., 2019). Because they bypass the surface glycoproteins targeted by NAIs, CEN inhibitors maintain potent antiviral efficacy against NAI-resistant strains (Yang, 2019). Furthermore, because CEN inhibitors potently suppress viral replication at the initial stage of transcription, preclinical studies have shown that they maintain significant protective efficacy even with delayed administration, thereby highlighting their potential to extend the therapeutic window (Fukao et al., 2019). Given their established clinical benefits, CEN inhibitors have been incorporated into recent treatment guidance; for example, the 2024 World Health Organization (WHO) influenza guidelines conditionally recommend baloxavir marboxil for patients with non-severe influenza at high risk of progression to severe disease (World Health Organization, 2024). Pixavir marboxil (TG-1000) is a novel CEN inhibitor designed to leverage this mechanism as a single-dose oral therapy for influenza.

To optimize its oral bioavailability and systemic exposure, pixavir marboxil was specifically designed as a prodrug that undergoes rapid, esterase-mediated *in vivo* hydrolysis to its active metabolite, pixavir, the moiety responsible for its anti-influenza activity. Although early clinical evaluation has confirmed a favorable safety, tolerability, and pharmacokinetic profile of this novel prodrug in healthy adults (Xu et al., 2023), its absorption, metabolism, and excretion (AME) characteristics in humans are not yet clear, which is crucial for clinical translation. Elucidating the pharmacokinetic disposition of a new chemical entity is a fundamental regulatory requirement to guide its clinical application (FDA, 2024). Human radiolabeled mass balance studies provide essential quantitative data for determining primary clearance mechanisms, quantifying absolute excretion pathways, and verifying comprehensive metabolic characteristics (FDA, 2024). In addition, characterizing the relative contributions of renal or non-renal (such as fecal) clearance pathway provides an essential basis for understanding the disposition profile of a new drug and informing subsequent clinical pharmacology evaluation (FDA, 2024).

This mass balance study was conducted to characterize the AME profiles, including mass balance, excretion pathways, and pharmacokinetics of [^14^C]pixavir marboxil in healthy Chinese male participants, and to provide a pharmacokinetic basis for its clinical use.

## 2 Materials and methods

### 2.1 Study design and participants

This was a phase I, single-center, open-label, non-randomized, single-dose mass balance study conducted in healthy Chinese male participants at Shanghai Xuhui Central Hospital (Shanghai, China). The study was conducted in accordance with the ethical principles of the Declaration of Helsinki and the International Council for Harmonisation Good Clinical Practice (ICH GCP) guidelines. The study was registered with the Drug Clinical Trial Registration and Information Disclosure Platform of the National Medical Products Administration (NMPA) under registration number CTR20231822. The protocol and informed consent form were reviewed and approved by the Ethics Committee of Shanghai Xuhui Central Hospital. All participants provided written informed consent prior to any study-specific procedures. Eligible participants were healthy males aged 18–40 years, with a body mass index (BMI) of 19.0–26.0 kg/m^2^ and regular bowel movements. General health was confirmed by normal or clinically insignificant findings in routine medical evaluations, including medical history inquiry, physical examinations, vital signs, 12-lead electrocardiograms (ECG), chest X-ray, and clinical laboratory tests. Key exclusion criteria included a history of any gastrointestinal conditions that could alter drug absorption, distribution, metabolism, or excretion; positive virology screening; and a history of drug or alcohol abuse. To ensure radiation safety, participants with significant medical or occupational radiation exposure within 1 year prior to dosing were also excluded.

### 2.2 Dosing and sample collection

The administered [^14^C]pixavir marboxil formulation was prepared by Value Pharmaceutical Services Co., Ltd (Nanjing, China). The final formulation demonstrated a chemical purity of 99.98%, a radiochemical purity of 99.66%, and a specific activity of 2.49 μCi/mg. The carbon-14 label was specifically incorporated via the cyclopropanecarbaldehyde moiety, which was retained in the active metabolite pixavir (TG-0527) following hydrolysis. Radiolabeled [^14^C]pixavir marboxil was synthesized by Moravek Inc (Brea, California, United States). Each capsule contained the [^14^C]pixavir marboxil drug substance together with a polyvinyl caprolactam–polyvinyl acetate–polyethylene glycol graft copolymer (Soluplus), microcrystalline cellulose, mannitol, croscarmellose sodium, and magnesium stearate, filled into a hydroxypropyl methylcellulose (HPMC) shell. Further details of the capsule composition and its preparation are available from the corresponding author upon reasonable request. Following an overnight fast of at least 10 h, participants received a single oral dose of approximately 40 mg (100 μCi) of [^14^C]pixavir marboxil as capsules (two 20-mg capsules), administered with 240 mL of water. Water was restricted for 1 h before and 1 h after dosing, and participants continued to fast for 4 h post-dose. Based on drug preparation and residual measurements after dosing, the actual average administered dose for the six participants was 38.3 mg (95.5 μCi).

Biological sample collection was guided by interim radioactivity monitoring based on quantitative termination criteria: blood sampling was discontinued when radioactivity concentrations fell below 3 times the pre-dose background level, whereas excreta collection ceased when the total recovery of radioactivity in urine and feces exceeded 80% of the dose, with less than 1% of the dose recovered in two consecutive collection intervals. Accordingly, the actual collection schedules were determined as follows: blood samples for pharmacokinetic and metabolism analyses were collected pre-dose (within 1 h) and at 1, 2, 3, 3.5, 4, 5, 6, 8, 12, 24, 48, 72, 120, 168, 192, 216, and 240 h post-dose; the collection was extended to 288 h for all participants, with further extension to 336 h for four participants. Blood samples dedicated to whole blood total radioactivity determination were collected pre-dose (within 1 h) and at 4, 8, 12, 24, and 48 h post-dose; additionally, extra samples for biotransformation profiling were collected at these same post-dose time points. Blank urine and fecal samples were collected randomly pre-dose (−24 to 0 h). Post-dose, excreta were collected continuously; urine was collected at intervals of 0–4, 4–8, 8–12, and 12–24 h during the first 24 h, followed by 24-h intervals up to 240 h. Fecal samples were collected at 24-h intervals throughout the post-dose period, with the actual collection duration ranging from 312 h to 384 h across all participants to ensure complete recovery of the administered dose.

### 2.3 Radioactivity and pharmacokineticanalysis

The total radioactivity in plasma and urine samples was determined directly by liquid scintillation counting (LSC) after mixing with a scintillation cocktail. For whole blood and homogenized fecal samples, aliquots were combusted using an automated sample oxidizer to trap the generated ^14^CO_2_ prior to LSC.

Plasma concentrations of the prodrug pixavir marboxil (TG-1000), its active metabolite pixavir (TG-0527), and the further conjugated metabolite TG-0600771 were quantified using a validated liquid chromatography-tandem mass spectrometry (LC-MS/MS) method. The calibration ranges were 1.00–300 ng/mL for pixavir marboxil, 2.00–600 ng/mL (high calibration curve range) and 0.100–10.0 ng/mL (low calibration curve range) for pixavir, and 0.400–400 ng/mL for TG-0600771, with corresponding lower limits of quantification (LLOQ) of 1.00, 2.00, 0.100 and 0.400 ng/mL, respectively. The precision (coefficient of variation, %CV) was ≤11.92% at the LLOQ and ≤7.14% at all other quality control levels. The overall accuracy (relative error, %RE) ranged from −1.30% to 10.63% across all analytes. Pharmacokinetic (PK) parameters for total radioactivity, pixavir marboxil, pixavir, and TG-0600771 were calculated by non-compartmental analysis (NCA) using Phoenix WinNonlin software (version 8.3, Certara). The whole blood-to-plasma radioactivity concentration ratio (K_b/p_) was calculated to evaluate the distribution of drug-related material into red blood cells.

For mass balance analysis, cumulative excretion of radioactivity in urine and feces over each collection interval was calculated as a percentage of the administered radioactive dose. Descriptive statistics for PK parameters and mass balance data were generated using SAS software (version 9.4, SAS Institute Inc.).

### 2.4 Metabolite profiling and identification

To generate representative metabolite profiles, plasma samples across different time points were pooled using the area under the curve (AUC)-proportional pooling method. Urine and fecal samples were pooled proportionally based on the sample volume and weight collected at each interval, respectively. Subsequently, these pooled plasma, urine, and homogenized fecal samples from participants were extracted with organic solvents, concentrated, and reconstituted. The samples were separated using a Shimadzu CBM20 A high-performance liquid chromatography (HPLC) system (Shimadzu, Japan). Chromatographic separation was achieved on an ACE Excel 3 C18 column (150 mm × 4.6 mm i. d.) protected by a Phenomenex SecurityGuard™ C18 cartridge (4.0 × 3.0 mm i. d.), maintained at 40 °C. The HPLC eluents were collected into Deepwell LumaPlate™-96 plates using an automated fraction collector at 15-s intervals. Radioactivity in each fraction was measured using a microplate counter, and off-line radio-chromatograms were generated using ARC Convert and Evaluation software (version 3.0.2.379). The radio-chromatograms were integrated to determine the peak area of each radioactive metabolite. These data were utilized to calculate the percentage of major metabolites relative to the total plasma radioactivity exposure (%AUC) or the administered dose (%Dose) excreted in urine and feces.

Major radioactive metabolites in plasma, urine, and feces were identified using liquid chromatography coupled with an online radioactivity monitor and high-resolution mass spectrometry (LC/RAM/MS). The analytical system consisted of a Shimadzu CBM20 A HPLC system, a Model 3 ARC™ online radioactivity monitor (AIM Research Co., United States), and a Q Exactive high-resolution mass spectrometer (Thermo Fisher Scientific, United States). The chromatographic conditions were identical to those described for the metabolite profiling.

### 2.5 Safety and tolerability evaluation

Safety and tolerability were assessed throughout the study by monitoring adverse events (AEs), clinical laboratory tests (hematology, blood biochemistry, coagulation, and urinalysis), 12-lead electrocardiograms (ECGs), vital signs, and physical examinations. All AEs were coded using the Medical Dictionary for Regulatory Activities (MedDRA) version 26.0 and graded for severity according to the Common Terminology Criteria for Adverse Events (CTCAE) version 5.0. The causal relationship between each AE and the study drug was assessed by the investigator on a case-by-case basis, considering the temporal relationship to dosing, the participant’s baseline clinical status, biological plausibility, the clinical course, and possible alternative causes. Causality assessment was based on the investigator’s individualized clinical judgment, consistent with the single-arm, open-label design of this human AME study.

### 2.6 Statistical analysis

Statistical analyses were primarily descriptive. The safety set (SS) included all participants who received the study drug and had at least one safety assessment. The pharmacokinetic concentration set (PKCS) and pharmacokinetic parameter set (PKPS) included participants with at least one valid post-dose concentration and evaluable PK parameter, respectively. The mass balance analysis set (MBS) included participants who provided at least one excreta sample with measurable radioactivity.

Descriptive statistics (e.g., mean, standard deviation [SD], coefficient of variation [CV%], median, minimum, and maximum) were used to summarize participant demographics, safety data, and PK parameters. PK parameters were estimated using non-compartmental analysis (NCA) with Phoenix WinNonlin software (version 8.3). For drug concentrations and key PK parameters, the geometric mean and geometric CV% were additionally calculated. The ratio of total radioactivity in whole blood to plasma (K_b/p_) was calculated as the mean and SD for each time point. Excretion data, including the percentage of administered radioactivity recovered in excreta per interval and cumulative excretion, were calculated using Microsoft Office Excel 2010. Other statistical summaries and calculations were performed using SAS software, version 9.4.

## 3 Results

### 3.1 Participant characteristics and safety

Six healthy Chinese male participants were enrolled and completed the study. The mean (±SD) age, height, weight, and BMI were 27.7 ± 3.01 years, 168.92 ± 4.92 cm, 62.15 ± 0.771 kg, and 21.83 ± 1.188 kg/m^2^, respectively. All participants were of Asian descent. None of the participants had a history of smoking, alcohol consumption, or drug abuse.

A single oral dose of approximately 40 mg (100 μCi) of [^14^C]pixavir marboxil was generally safe and well-tolerated. No deaths, serious adverse events (SAEs), or adverse events (AEs) leading to study discontinuation were reported. Three participants (50.0%) experienced a total of three treatment-emergent adverse events (TEAEs), all of which were Grade 1 (mild) in severity and resolved spontaneously without medical intervention. These included elevated blood triglycerides (1 participant, 16.7%; peak value, 3.24 mmol/L; reference range, 0.1–1.8 mmol/L), which was considered possibly related to the study drug, as well as elevated urine leukocytes (1 participant, 16.7%; peak value, 35–40/HP; reference range, negative) and diarrhea (1 participant, 16.7%), both of which were considered unlikely to be related to the study drug.

### 3.2 Pharmacokinetics

**FIGURE 1:**
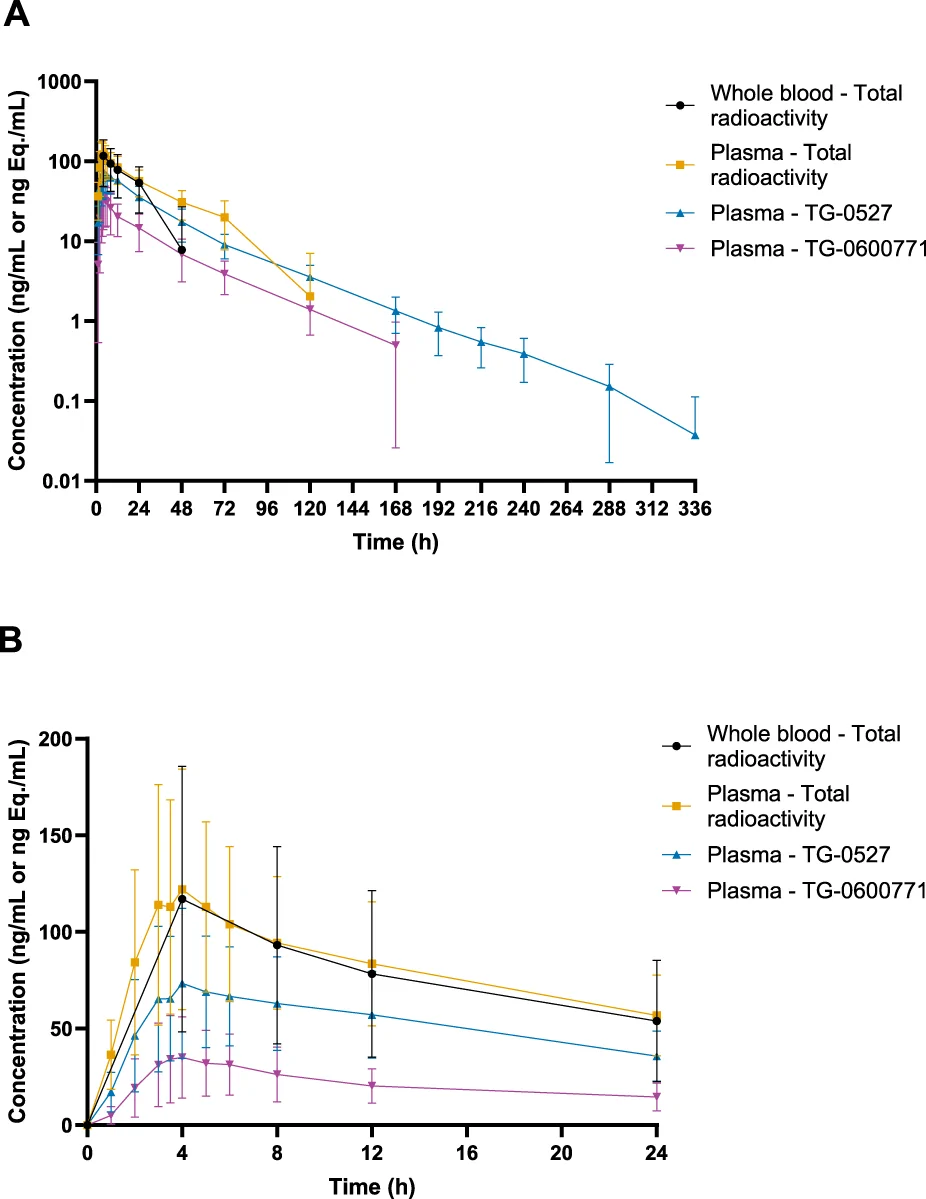
Mean concentration-time profiles of total radioactivity in whole blood and plasma, and metabolites (pixavir (TG-0527) and TG-0600771) in plasma following a single oral dose of 40 mg (100 μCi) [^14^C]pixavir marboxil in six healthy Chinese male participants. **(A)** Semi-logarithmic profile up to 336 h; **(B)** Expanded linear profile from 0 to 24 h. Note: Data are presented as mean + standard deviation (N = 6).

Following a single oral dose of 40 mg (100 μCi) [^14^C]pixavir marboxil (TG-1000), the unchanged prodrug pixavir marboxil was undetectable in plasma. The mean semi-logarithmic concentration-time profiles of total radioactivity in whole blood and plasma, pixavir, and TG-0600771 are presented in Figure 1, and their key pharmacokinetic parameters are summarized in Table 1.

**TABLE 1:** Pharmacokinetic parameters of total radioactivity and metabolites (pixavir (TG-0527) and TG-0600771) in plasma following a single oral dose of 40 mg (100 μCi) [^14^C]pixavir marboxil in healthy Chinese male participants (N = 6).

Parameter	Unit	Total radioactivity	Pixavir (TG-0527)	TG-0600771
T_max_	h	4.51 (3.00, 6.00)	4.51 (4.00, 6.00)	4.51 (3.50, 6.00)
C_max_	ng Eq./mL or ng/mL	115 (65.6)	70.7 (62.2)	30.7 (74.3)
AUC_0-t_	h·ng Eq./mL or h·ng/mL	3,310 (61.4)	2,480 (46.8)	945 (55.8)
$\text{AU}C_{0‐\infty }$	h·ng Eq./mL or h·ng/mL	4,220 (51.5)	2,490 (46.8)	981 (54.7)
t_1/2_	h	31.4 ± 4.85	41.6 ± 8.04	32.0 ± 5.51
MRT_0-t_	h	26.0 ± 4.68	40.5 ± 4.39	35.2 ± 5.74
Vd/F	L	425 (48.3)	NA	NA
CL/F	L/h	9.48 (51.5)	NA	NA
Metabolite/Total AUC ratio^a^	%	NA	70.49	19.99

Abbreviations: AUC_0-t_, area under the concentration-time curve from time zero to the last measurable concentration; ${AUC}_{0-\infty }$ , area under the concentration-time curve from time zero to infinity; C_max_, maximum observed concentration; CV, coefficient of variation; MRT, mean residence time; NA, not applicable; t1/2, terminal half-life; T_max_, time to maximum concentration.

Data are presented as geometric mean (geometric CV%) for C_max_, AUC, Vd/F, and CL/F; arithmetic mean ± standard deviation for t1/2 and MRT_0-t_; and median (minimum, maximum) for T_max_. Units for total radioactivity are expressed as equivalents (Eq.).

a The metabolite to total radioactivity AUC ratio (%) was calculated as [(Metabolite ${AUC}_{0-\infty }$ /Metabolite MW)/(Total radioactivity ${AUC}_{0-\infty }$ /Prodrug MW)] × 100, where the molecular weights (MW) are 540.54 for pixavir marboxil (TG-1000), 452.47 for pixavir (TG-0527), and 628.60 for TG-0600771.

The total radioactivity, pixavir, and TG-0600771 reached their maximum concentrations synchronously, with a median T_max_ of 4.51 h for all three analytes (overall range 3.00–6.00 h). The geometric mean (CV%) C_max_ values were 115 ng Eq./mL (65.6%) for total radioactivity, 70.7 ng/mL (62.2%) for pixavir, and 30.7 ng/mL (74.3%) for TG-0600771. The geometric mean (CV%) ${AUC}_{0-\infty }$ was 4,220 hng Eq./mL (51.5%) for total radioactivity, 2,490 hng/mL (46.8%) for pixavir, and 981 hng/mL (54.7%) for TG-0600771. The two major circulating metabolites, pixavir and TG-0600771, accounted for 70.49% and 19.99% of the total plasma radioactivity ${AUC}_{0-\infty }$ (estimated by LC-MS/MS), respectively.

The apparent terminal half-lives (t_1/2_) for total radioactivity, pixavir, and TG-0600771 were 31.4 ± 4.85 h, 41.6 ± 8.04 h, and 32.0 ± 5.51 h, respectively. Furthermore, the individual whole blood-to-plasma total radioactivity concentration ratios ranged from 0.881 to 1.20, with mean values across different sampling time points ranging from 0.912 to 1.06. These ratios remained close to 1.0 across all time points. The geometric mean apparent volume of distribution (Vd/F) and apparent clearance (CL/F) for total radioactivity were 425 L (CV% 48.3%) and 9.48 L/h (CV% 51.5%), respectively.

### 3.3 Mass balance and excretion

**FIGURE 2:**
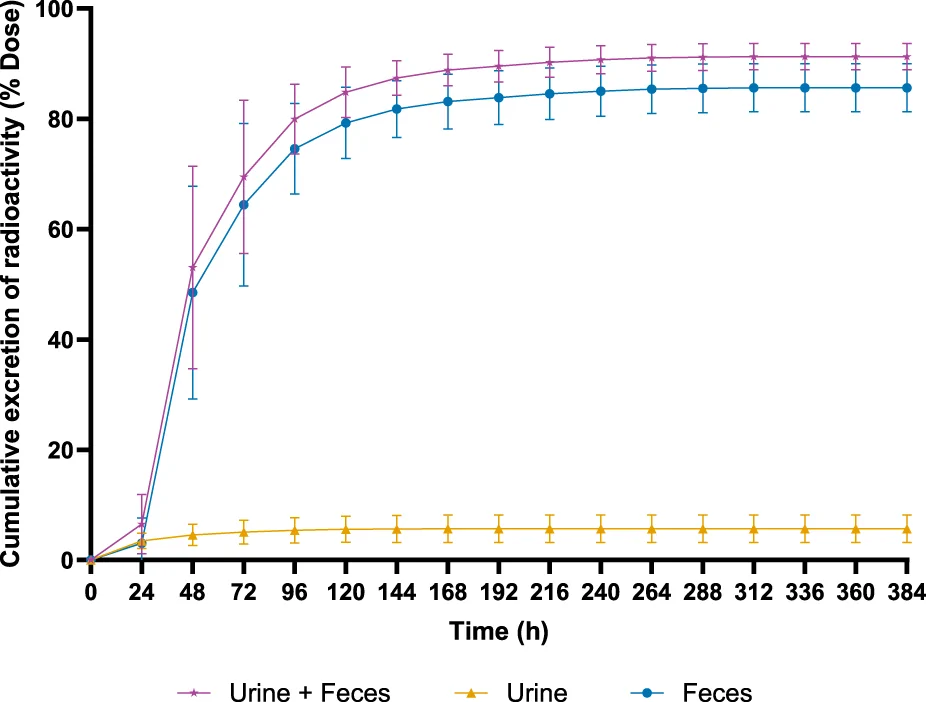
Mean cumulative excretion of total radioactivity in urine and feces following a single oral dose of 40 mg (100 μCi) [^14^C]pixavir marboxil in six healthy Chinese male participants. Note: Data are presented as mean +standard deviation (N = 6).

Following a single oral dose of 40 mg (100 μCi) [^14^C]pixavir marboxil, the administered radioactivity was recovered in feces and urine. The cumulative excretion of total radioactivity in urine and feces over the 384-h collection period is depicted in Figure 2. The overall mean mass balance (total recovery of administered radioactivity) was 91.29% ± 2.39%. A total of 85.62% ± 4.35% of the administered radioactive dose was recovered in feces, and 5.68% ± 2.48% was recovered in urine. The excretion of total radioactivity occurred rapidly, with 84.82% of the administered dose recovered within the first 120 h post-dose. Mass balance (>90% recovery) was achieved at 216 h post-dose, with a cumulative recovery of 90.22%. Excretion of radioactivity was essentially complete by 168 h post-dose, after which the daily excretion fell below 1% of the administered dose.

### 3.4 Metabolite profiling and identification

Intact pixavir marboxil was not detected in plasma and urine and accounted for 0.27% of the administered dose in feces. The detailed distribution of the prodrug and its metabolites in plasma, urine, and feces is summarized in Table 2. In pooled plasma, the predominant circulating drug-related components were the active metabolite pixavir (TG-0527) and its glucuronide conjugate TG-0600771, representing 70.10% and 29.02% of the total plasma radioactivity exposure (determined by radiochemical detection), respectively. Together, the identified radioactive peaks accounted for 99.12% of the total plasma radioactivity.

**TABLE 2:** Proportions of [^14^C]pixavir marboxil and its major metabolites relative to total plasma radioactivity exposure (%AUC) and administered dose in excreta (%Dose).

Drug‐related component	Plasma (%AUC)	Urine (%Dose)	Feces (%Dose)	Total excretion (%Dose)
Pixavir marboxil (prodrug)	ND	ND	0.27	0.27
Pixavir (M452)	70.10	0.31	72.45	72.76
TG-0600771 (M628-2)	29.02	2.51	ND	2.51
M468-1	ND	0.37	3.37	3.74
M468-2	ND	0.39	3.32	3.71
M628-1	ND	0.17	ND	0.17
Subtotal (uncharacterized)	0.88	1.93	6.21	8.13
Subtotal (identified)	99.12	3.75	79.41	83.16
Total radioactivity	100	5.68	85.62	91.29

*ND, not detected. %AUC, represents the percentage of the specific analyte relative to the total plasma radioactivity exposure. Excretion data (%Dose) represent the cumulative amount recovered over 0 to 384 h post-dose, expressed as a percentage of the administered radioactive dose. Subtotal (Uncharacterized) refers to the sum of individual radioactive peaks that accounted for < 5.00% of plasma AUC, or < 3.00% of the administered dose.*

**FIGURE 3:**
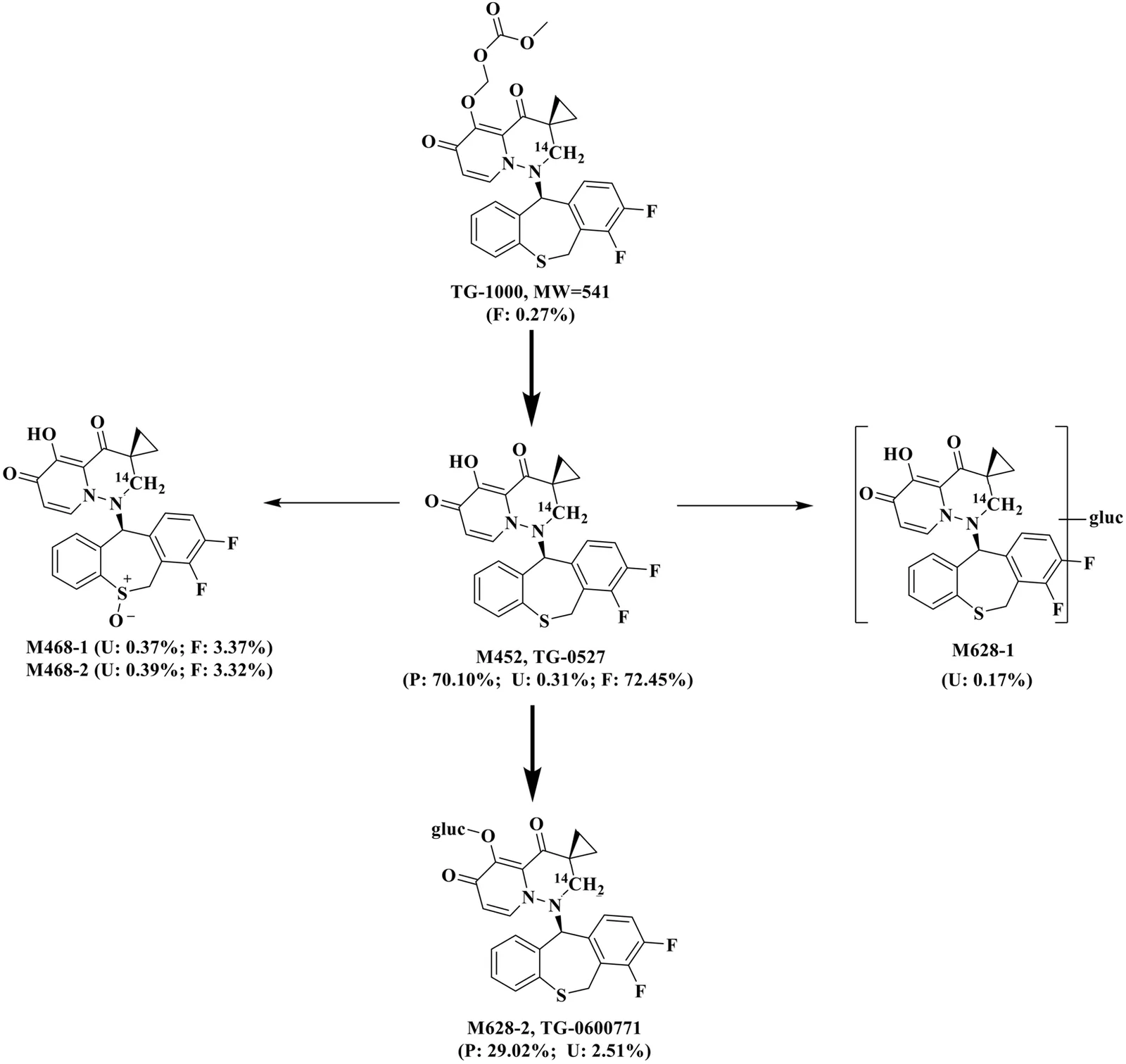
Proposed metabolic pathways of [^14^C]pixavir marboxil in six healthy Chinese male participants. Notes: ^14^C indicates the position of the carbon-14 radiolabel. Pixavir marboxil and pixavir correspond to TG-1000 and TG-0527 (M452), respectively. P represents plasma, expressed as a percentage of total radioactivity exposure (%AUC). U and F represent urine and feces, respectively, expressed as a percentage of the administered dose (%Dose).

The predominant fecal metabolite was pixavir, accounting for 72.45% of the administered dose. TG-0600771 was the primary urinary metabolite, accounting for 2.51% of the dose. In addition to the unchanged prodrug, a total of five metabolites were identified (Figure 3). Alongside the major metabolites pixavir and TG-0600771, minor metabolites including sulfoxide products (M468–1 and M468-2) and another glucuronide conjugate (M628-1) were also identified in excreta, each accounting for a small fraction of the dose. Across all excreta, pixavir and TG-0600771 accounted for 72.76% and 2.51% of the administered dose, respectively. The chemical structures of these major circulating and excreted metabolites were further confirmed by high-resolution mass spectrometry (HR-MS). Structural elucidation of the metabolites was performed using LC-HRMS based on accurate mass and fragmentation patterns. For instance, the major active metabolite pixavir (M452) exhibited a protonated molecular ion [M + H]^+^ at m/z 453.1079. Its glucuronide conjugate TG-0600771 (M628-2) showed an [M + H]^+^ at m/z 629.1400, which is 176 Da higher than that of pixavir, with a characteristic MS/MS fragment at m/z 453.1074 indicating the loss of the glucuronic acid moiety. Similarly, the sulfoxide metabolites M468–1 and M468-2 both displayed [M + H]^+^ at m/z 469.1028, representing a 16 Da mass shift from pixavir due to mono-oxidation.

## 4 Discussion

This study provides the first comprehensive characterization of the mass balance and excretion pathways of a single oral dose of [^14^C]pixavir marboxil in six healthy Chinese male participants. The administered radioactive dose was well-recovered, with a cumulative mean recovery of 91.29% in total excreta (urine and feces combined), confirming the reliability of the mass balance assessment. Although a population pharmacokinetic and exposure–response analysis of oral pixavir marboxil in adults and adolescents has been reported before, in which glucuronidation of the active metabolite was identified as a metabolic pathway (Yang et al., 2026), such model-based analyses cannot define the absolute disposition of the drug. The present radiolabeled study is therefore complementary rather than overlapping: it quantifies the absolute excretion routes and the relative contributions of renal and non-renal clearance, and it structurally confirms the circulating and excreted metabolites by high-resolution mass spectrometry—information that cannot be derived from population modeling alone. Furthermore, the radioactivity was predominantly recovered in feces rather than urine, demonstrating that the drug is primarily eliminated via non-renal pathways. Regarding systemic exposure, intact pixavir marboxil was undetectable in plasma, while its active metabolite, pixavir, and subsequent metabolites accounted for nearly all systemic radioactivity. The absence of the intact prodrug, combined with the synchronous T_max_ of the analytes, indicates that pixavir marboxil was rapidly absorbed, undergoing complete conversion into its active metabolite, pixavir, during absorption or presystemic first-pass metabolism. Furthermore, the elimination of these drug-related components was relatively slow. The whole blood-to-plasma radioactivity ratios remained close to 1.0, which suggests a certain degree of binding of the drug-related materials to red blood cells.

Although prodrugs are often engineered with high lipophilicity to enhance oral absorption, slow systemic cleavage may lead to undesired extensive tissue distribution and off-target toxicity of inactive precursors (Huttunen et al., 2011; Rautio et al., 2018). The rapid esterase-mediated conversion of pixavir marboxil prevents prolonged systemic circulation of the lipophilic precursor, thereby facilitating a predictable and stable systemic exposure to the active antiviral metabolite.

Overall, metabolic profiling indicates that pixavir marboxil undergoes rapid enzymatic hydrolysis to form the active metabolite pixavir. Subsequently, pixavir is further metabolized via uridine 5′-diphospho-glucuronosyltransferase (UGT)-mediated glucuronidation, alongside minor oxidative pathways. Collectively, this primary metabolic and elimination cascade accounts for at least 75.27% of the administered dose recovered in excreta, resulting in pixavir and its conjugate TG-0600771 constituting the vast majority of circulating drug-related exposure. Of note, the slightly higher proportion of TG-0600771 determined by direct radio-chromatogram peak integration (29.02%) compared to LC-MS/MS estimates (19.99%) is typically attributed to differences in analytical methodologies and detection sensitivities, a discrepancy commonly observed in mass balance studies. Importantly, no major cytochrome P450 (CYP)-mediated oxidative metabolites were identified in plasma, indicating that the clearance of pixavir is largely independent of the CYP enzyme system. This non-CYP-dependent metabolic profile reduces the likelihood that pixavir acts as a victim of CYP-mediated drug-drug interactions (DDIs) when co-administered with other agents (Ozbey et al., 2024). It should be emphasized, however, that a low potential to act as a DDI victim does not in itself preclude a perpetrator role; *in vitro*, pixavir showed neither inhibition nor induction of the major CYP enzymes (unpublished data). In clinical practice, influenza patients commonly use acetaminophen for fever management and may require macrolide antibiotics for potential secondary bacterial infections (World Health Organization, 2009; Lee et al., 2017). Both acetaminophen and macrolide antibiotics are heavily associated with CYP450-related risks, such as enzyme inhibition or toxic metabolite formation (Tornio et al., 2019; Mazaleuskaya et al., 2015). By relying on UGT-mediated conjugation rather than CYP-mediated oxidation, pixavir avoids metabolic competition and inhibition within the CYP system, thereby theoretically reducing the risk of DDIs in these common combination therapies. Furthermore, because pixavir marboxil is administered as a single oral dose for influenza treatment, transient pharmacokinetic perturbations from concomitant medications would be less likely to be amplified by repeated dosing or cumulative exposure.

Following hepatic biotransformation, fecal excretion was the predominant route of elimination for drug-related material (85.62%), whereas renal excretion represented a minor pathway (5.68%). In particular, urinary excretion of the pharmacologically active metabolite pixavir accounted for only 0.31% of the administered dose. Although the glucuronide conjugate TG-0600771—a major circulating metabolite and the principal drug-related species in urine—could in principle undergo enterohepatic recirculation and be reconverted to the active moiety, the low urinary recoveries of total radioactivity and active pixavir further support a predominantly non-renal disposition of active pixavir (EMA, 2015).

The clinical relevance of these AME characteristics is further highlighted when compared with existing anti-influenza therapies. Within the class of PA endonuclease inhibitors, pixavir marboxil—approved by the NMPA in December 2025—shares similar non-CYP-mediated metabolic pathways (mainly UGT) and predominantly non-renal excretion with baloxavir marboxil (FDA. XOFLUZA, 2025). Furthermore, a recent phase I clinical study of the newly approved PA inhibitor seloxavir marboxil (ZX-7101 A) has also demonstrated that its exposure is not significantly affected by strong CYP3A4 inhibitors (with AUC increasing by only 58.0% when co-administered with itraconazole, requiring no dose adjustment), indicating a CYP-independent metabolic pathway (Wu et al., 2025). However, avoiding CYP-mediated metabolism is a specific structural characteristic rather than an absolute class effect, as clinical evaluations of another PA inhibitor, suraxavir marboxil (GP681), have confirmed that its active metabolite is primarily metabolized by CYP3A4 (resulting in an approximately 2.5-fold increase in AUC when co-administered with itraconazole), thereby introducing potential drug-drug interactions (Han et al., 2025). When examining inhibitors of other targets, distinct pathways are observed. The PB1 inhibitor favipiravir is primarily eliminated via aldehyde oxidase–mediated metabolism, with its inactive metabolite (T-705M1) excreted renally (Du and Chen, 2020). The active components of oseltamivir and peramivir, both neuraminidase inhibitors (NAIs), are predominantly cleared by the kidneys (e.g., >90% recovered in urine for oseltamivir) (Robson et al., 2006; Wester and Shetty, 2016). Additionally, the hemagglutinin (HA) inhibitor arbidol undergoes extensive CYP3A4-driven metabolism (Deng et al., 2013).

Considering the overall findings, pixavir is not primarily eliminated through CYP-mediated pathways and exhibits low renal excretion of the inactive metabolite. Together with the single-dose oral regimen for influenza treatment, these characteristics suggest a low potential for clinically meaningful CYP-mediated DDIs and for renal-excretion-related accumulation of the inactive metabolite.

A limitation of this study is the relatively small sample size (N = 6). Although this sample size is consistent with guidance on human radiolabeled mass balance studies (FDA, 2024), it limits broad generalizability. Enrollment was restricted to healthy male participants in accordance with common practice for such studies (Ramamoorthy et al., 2022), primarily owing to radiation-safety and reproductive-toxicity considerations associated with administering a radiolabeled compound to women of childbearing potential, as well as the practical considerations of complete excreta collection. Although our population pharmacokinetic analysis of pixavir (Yang et al., 2026) and the prescribing information for the cap-dependent endonuclease inhibitor baloxavir marboxil (FDA. XOFLUZA, 2025) did not identify clinically meaningful sex-related differences in pharmacokinetic exposure, the present study was not designed to directly evaluate sex-related differences in mass balance, excretion, or metabolite profiles. All participants were Han Chinese; therefore, extrapolation of these findings to other ethnic populations should be made with appropriate caution.

In conclusion, this study demonstrates that pixavir marboxil undergoes complete presystemic conversion to its active metabolite, pixavir, followed by UGT-mediated metabolism and predominant fecal excretion. The limited renal excretion of active pixavir and the absence of major CYP-mediated oxidative metabolites suggest a low likelihood of renal-excretion-related accumulation of the inactive metabolite and of pixavir acting as a victim of clinically meaningful CYP-mediated drug-drug interactions. These findings support a well-characterized and favorable disposition profile of pixavir marboxil.

## Data Availability

The original contributions presented in the study are included in the article/supplementary material, further inquiries can be directed to the corresponding author.
